# Flexico: An efficient dual-mode consensus protocol for blockchain networks

**DOI:** 10.1371/journal.pone.0277092

**Published:** 2022-11-03

**Authors:** Shuyang Ren, Choonhwa Lee, Eunsam Kim, Sumi Helal

**Affiliations:** 1 Department of Computer Science, Hanyang University, Seoul, Korea; 2 Department of Computer Engineering, Hongik University, Seoul, Korea; 3 CISE Department, University of Florida, Gainesville, FL, United States of America; Jazan University Faculty of Computer Science, SAUDI ARABIA

## Abstract

Blockchain is a Byzantine fault tolerant (BFT) system wherein decentralized nodes execute consensus protocols to drive the agreement process on new blocks added to a distributed ledger. Generally, two-round communications among 3f+1 nodes are required to tolerate up to f faults in BFT-based consensus networks. This communication pattern corresponds to the worse-case scenario of consensus achievement, even under asynchronous network conditions. Nevertheless, it is not uncommon for a network to operate under better conditions, where a consensus can be reached with a lower communication cost. Hence, with the addition of a faster optimistic path toward an agreement, the idea of dual-mode consensus has been proposed as a promising approach to enhance the performance of asynchronous BFT protocols. However, this opportunity is not completely exploited by existing dual-mode protocols as the fast path can be followed only in a nonfaulty and synchronous network. This article presents a novel dual-mode protocol consisting of fast and backup subprotocols. To create different consensus committees for fast and backup-mode operations, the network contains both active and passive nodes. A consensus can be expedited through a fast-mode operation when majority of the active nodes can communicate synchronously. Under non-ideal conditions, the backup protocol takes over the agreement process from its fast-mode counterpart without starting over the suspended round. The safety and liveness of the proposed protocol are guaranteed with lower communication costs, which balance the trade-off between protocol efficiency and availability.

## Introduction

Over the years, replication technologies have been widely used in distributed systems to ensure consistency and fault tolerance. Particularly, replication technologies can be divided into active and passive replications [1]. In a passive replication, a client request is processed by the primary node, and the result is transferred to other replicas. In an active replication, also known as a state machine replication (SMR), the same request is processed by all replicas. In recent years, Byzantine fault tolerant (BFT) SMR protocols have been increasingly employed as consensus algorithms in decentralized and tamper-resistant blockchain systems. Following the success of cryptocurrencies, blockchain technologies have also been used in the Internet of Things devices, smart homes, healthcare, and other areas of application [2–10]. Correspondingly, BFT protocols have attracted considerable attention in academia and industry. Such protocols can tolerate Byzantine faults, where adversary nodes can exhibit arbitrary behaviors, making them vital components in maintaining the security of blockchain systems. However, unlike a standard SMR use case, blockchain systems mostly comprise large-scale nodes, which present new challenges to the implementation of BFT protocols.

To understand this problem, we must consider the operation of BFT protocols. Let us consider the classic practical BFT (PBFT) algorithm, an asynchronous BFT algorithm widely used in private blockchains, as an example [11]. To tolerate Byzantine nodes, 3f+1 nodes must run a consensus algorithm comprising three phases: pre-preparation, preparation, and commitment phases. In the pre-preparation phase, the leader node multicasts a pre-prepared message to other nodes. After receiving the pre-prepared message, each node broadcasts a prepared message to other nodes during the preparation phase. Similarly, when each node receives the prepared message, it broadcasts a commitment message during the commitment phase to complete the consensus processing. However, this all-to-all communication mode increases the communication complexity to $O(n^{2})$, which increases the number of messages exponentially with increasing number of nodes. Consequently, BFT protocols are unsuitable for blockchain systems composed of large-scale nodes and are limited to small-scale private blockchain systems. Therefore, increasing number of studies have focused on reducing communication costs and improving the efficiency of the applied protocols.

A synchronous network environment is undoubtedly beneficial for establishing efficient consensus protocols because only two phases, i.e., preparation and commitment, are required for a synchronous consensus protocol. This lowers the communication cost, thereby improving the efficiency of the protocol. However, robustness against asynchronous networks is critical in actual blockchain systems. Bearing this in mind, a set of dual-mode protocols that provide an optimistic fast-path protocol in a synchronous network environment, as well as a backup protocol in an asynchronous network environment, is typically adopted [12–15]. The original objective of this idea is to obtain a highly efficient synchronous consensus protocol while ensuring robustness against asynchronous networks. Among the related protocols, fast and efficient protocols need to be executed in a purely synchronous and error-free system. However, uncertain network delays are common in real-world systems, and thus, fast protocols are generally useless; consequently, the protocols rely heavily on backup protocols. In addition, dual-mode protocols usually apply fast protocols by default and require a view change to switch to the backup protocol, thus increasing the cost associated with backup protocol running compared with the standard BFT approach.

To address the issues of dual-mode protocols mentioned above, in this paper, we propose a new dual-mode protocol that can reach a consensus with maximum use of the fast protocol. The key concept behind this approach consists of two parts. First, we divide the system nodes into active and passive nodes. By default, the system allows active nodes to take lead while running a fast protocol. At this time, passive nodes do not participate in the consensus processing but update the state according to the consensus results obtained from the active nodes. When the active nodes fail to successfully complete the consensus process owing to an unstable network delay, the system switches to the backup protocol. At this point, the passive nodes participate in the consensus process, continuing the execution of the fast protocol left incomplete by the active nodes without the need for a view change. This provides the advantage of maintaining the robustness of the consensus protocol against asynchronous networks while improving the efficiency of the fast protocol. Moreover, our consensus protocol can seamlessly switch between the fast protocol and backup protocol without incurring additional communication costs. Instead of restarting the unfinished consensus processing of the active nodes, the passive nodes simply take over the process, which implies that the messages collected by the nodes in the default fast protocol are not wasted even if the system switches to the backup protocol. Second, we relax the operating limitations of our fast protocol. Thus, our fast protocol can achieve consensus when more than half of the active nodes are error-free and communicate synchronously with each other. As an advantage of this change, in contrast to other dual-mode protocols that require the entire system to be purely synchronous and error-free, the condition for our fast protocol operation is significantly easier to satisfy in practice. Consequently, our consensus protocol can maximize the use of the fast protocol to complete consensus processing.

The key to realizing this approach lies in ensuring that the fast protocol can run when more than half of the active nodes are error-free and are able to communicate synchronously. Furthermore, messages exchanged while running the default fast protocol should be continually utilized after the system switches to the backup protocol. Therefore, based on our previous study we propose Flexico, a dual-mode protocol that relies on a t-of-n threshold Boneh-Lynn-Shacham (BLS) signature [16–18]. The threshold BLS signature scheme uses a distributed key generation (DKG) protocol to generate keys for the nodes and can tolerate arbitrary t<n/2 malicious parties [19]. In addition, for the same message M, any subset of nodes larger than t can generate an aggregated signature that can be verified based on a unique fixed-group public key. We use the threshold BLS signature scheme as a voting mechanism to verify the validity of the proposed blocks. Owing to the gossip communication model, blocks can reach a consensus after a single round of one-way communication, reducing the communication complexity associated with verification of the blocks to O(logn) [20].

This study contributes in terms of the following key aspects:

- We address the low utilization of fast protocols in existing dual-mode protocols. The conditions for running the fast protocol are more relaxed, which allows our protocol to run the fast protocol and reach consensus to the maximum extent possible.
- The system runs the fast protocol by default and switches to the backup protocol when the network becomes unstable. However, switching between these two modes does not require a change in view. The backup protocol overtakes the unfinished fast protocol, and the messages recorded in the fast protocol can still be utilized in the backup protocol.
- Our protocol combines the threshold BLS signature technique with the gossip communication model, reducing the communication complexity of the protocol to O(logn).
- Compared with the state-of-the-art SBFT protocol, our protocol achieves a 40% lower latency. In addition, our protocol increases the probability of achieving a consensus using its fast protocol to over 95%, which is greater than the corresponding 50% or lower probability of the SBFT protocol.

The remainder of this paper is structured as follows. In the next section, we discuss the technological background for this study. The overall design and detailed description of the protocol are presented in the section on the Flexico protocol. Subsequently, we present an analysis of the liveness of our fast-mode protocol and report the results of our performance evaluation. We then summarize previous related studies and describe the future scope of our research. Finally, certain concluding remarks regarding this study are provided.

## Background

Generally, a consensus protocol assumes realistic target networks wherein either crash or Byzantine faults can occur. Previous studies have proven that the achievement of a consensus is impossible in a purely asynchronous environment with a single faulty node [21]. Thus, to make the problem more tractable, certain protocols implement a consensus in synchronous networks, wherein a finite bound Δ for the message delivery is known to establish bounds for communication delays [22–24]. for the message delivery is known to establish bounds for communication delays. Therefore, to provide liveness, several BFT consensus protocols for asynchronous blockchain systems rely on partially synchronous networks [25]. In particular, two circumstances can be considered partially synchronous. For the first, an upper bound Δ exists but is unknown to the participants. For the second, Δ is known, but the bound holds after an unspecified finite time. Under a partially synchronous assumption, the system requires at least 3f+1 nodes and two rounds of message exchanges to tolerate Byzantine faults. The PBFT protocol is one of the most well-known consensus protocols that rely on partially synchronous time assumptions [11]. Subsequently, several BFT-compliant consensus protocols have been explored to improve the performance of BFT protocols. The characteristics of some representative protocols are summarized in Table 1. Because a BFT protocol is mostly leader-based, when a malicious node is elected as the leader, it negatively affects the performance of the protocol. Therefore, protocols such as Algorand, IBFT, and Prosecurtor [26–28] add a leader selection algorithm to avoid the selection of malicious nodes as leaders. In addition, although numerous BFT protocols maintain liveness based on partially synchronous time assumptions, HoneyBadger and other approaches [29–32] use consensus protocols developed to run in asynchronous networks, improving the availability of BFT protocols. Moreover, other approaches, including DAG-Rider and Tusk [33, 34] utilize a directed acyclic graph (DAG) structure to improve the performance of the BFT consensus protocol.

**Table 1:** Comparison of BFT-based consensus protocols.

Protocol	Adversary tolerate	Communication model	Communication complexity	Throughput	Latency	Finality
Algorand [26]	*f* < *n*/3	Patially synchronous	N/A	Medium	Medium	Instant
IBFT [27]	*f* < *n*/3	Patially synchronous	*O*(*n*^2^)	N/A	Low	Deterministic
Prosecutor [28]	*f* < *n*/3	Patially synchronous	*O*(*n*)	Medium	Low	Deterministic
HoneyBadger [29]	*f* < *n*/3	Asynchronous	*O*(*n*^2^)	Low	High	Instant
Dumbo1 [30]	*f* < *n*/3	Asynchronous	*O*(*n*^2^)	Medium	Medium	Instant
Dumbo2 [30]	*f* < *n*/3	Asynchronous	*O*(*n*^2^)	High	Medium	Instant
DispersedLedger [31]	*f* < *n*/3	Asynchronous	*O*(*n*)	High	Low	Instant
AleaBFT [32]	*f* < *n*/3	Asynchronous	*O*(*n*^2^)	Medium	High	Deterministic
DAG-Rider [33]	*f* < *n*/3	N/A	*O*(*n*)	N/A	N/A	N/A
Tusk [34]	*f* < *n*/3	N/A	*O*(*n*)	Very High	Low	N/A

To ensure good performance of BFT protocols, a synchronous network is undoubtedly the best operating environment because an unstable asynchronous network adds an additional communication burden on the consensus process. However, in practical large-scale networks, an unstable network latency is extremely common. It is, thus, also important for consensus algorithms to become robust against asynchronous networks. Therefore, certain protocols attempt to maintain improved performance in a synchronous network environment while also achieving robustness against asynchronous networks. Accordingly, several systems have adopted this idea by utilizing a dual-mode consensus protocol [12–15, 35, 36]. The characteristics of such protocols are summarized in Table 2. Particularly, dual-mode protocols consist of a fast optimistic subprotocol and a backup protocol. The optimistic fast protocol operates when the network state is synchronous, whereas the backup protocol tolerates unfavorable network conditions.

**Table 2:** Comparison of dual-mode consensus protocols.

Protocol	Fast-mode			Backup-mode			View change
	Adversary tolerate	Communication model	Communication complexity	Adversary tolerate	Communication model	Communication complexity	
Zyzzyva [14]	*f* = 0	Synchronous	*O*(*n*)	*f* < *n*/3	Patially synchronous	*O*(*n*^2^)	✓
Azyzzyva [15]	*f* = 0	Synchronous	*O*(*n*)	*f* < *n*/3	Patially synchronous	*O*(*n*^2^)	✓
CheapBFT [13]	*f* = 0	Synchronous	*O*(*n*^2^)	*f* < *n*/3	Patially synchronous	*O*(*n*^2^)	✓
Bolt-Dumbo [35]	*f* < *n*/3	Synchronous	*O*(*n*)	*f* < *n*/3	Asynchronous	*O*(*n*^2^)	✓
Jolteon-Ditto [36]	*f* < *n*/3	Synchronous	*O*(*n*)	*f* < *n*/3	Patially synchronous	*O*(*n*^2^)	✓
SBFT [12]	*f* = 0	Synchronous	*O*(*n*)	*f* < *n*/3	Patially synchronous	*O*(*n*)	✓

Although Byzantine adversaries can behave arbitrarily and delay communication, they cannot subvert cryptographic techniques used in message authentication. In advanced BFT protocols, although the use of cryptography is beyond such authentication, it is also an efficient strategy for collecting signatures as a voting approach to prove block validity among consensus-processing participants. Previous studies have developed an efficient and robust threshold signature scheme based on the BLS approach [17, 18]. Herein, participants secretly share their keys through an interactive DKG protocol, and the threshold signature can be verified using a unique corresponding public key [19]. Compared to other signature schemes, such as Rivest–Shamir–Adleman and Schnorr, the BLS signature has a significantly shorter length [37, 38]. In addition, the threshold BLS signature can be generated in a non-interactive manner with no extra overhead, rendering the scheme favorable for some consensus protocols, such as SBFT and FBFT [39].

## Flexico protocol

Herein, we introduce Flexico, a novel dual-mode consensus protocol that includes fast- and backup-mode protocols. Flexico is built on Concordia, an efficient BFT protocol based on a threshold BLS signature scheme and a gossip communication pattern.

### System model

We assume a standard partially synchronous BFT protocol model wherein a Byzantine adversary can control at most f nodes among all the 3f+1 nodes. Each node holds a pair of keys containing public and private keys. Moreover, we assign identities to participants in the system based on a peer-discovery algorithm, where the nodes are assumed to have network connectivity. The system divides time into epochs, that is, predefined periods. At each epoch e, we assume that we generate a random seed ${\text{Rnd}}_{e}$. After successfully establishing the identities, we use ${\text{Rnd}}_{e}$ as an input to the Fisher–Yates shuffle algorithm to compute a pseudo-random permutation $\pi_{e}$ of 1,2,...,n for the identities [40]. The permutation is further divided into two parts to identify the active and passive nodes.

**Fig 1:**
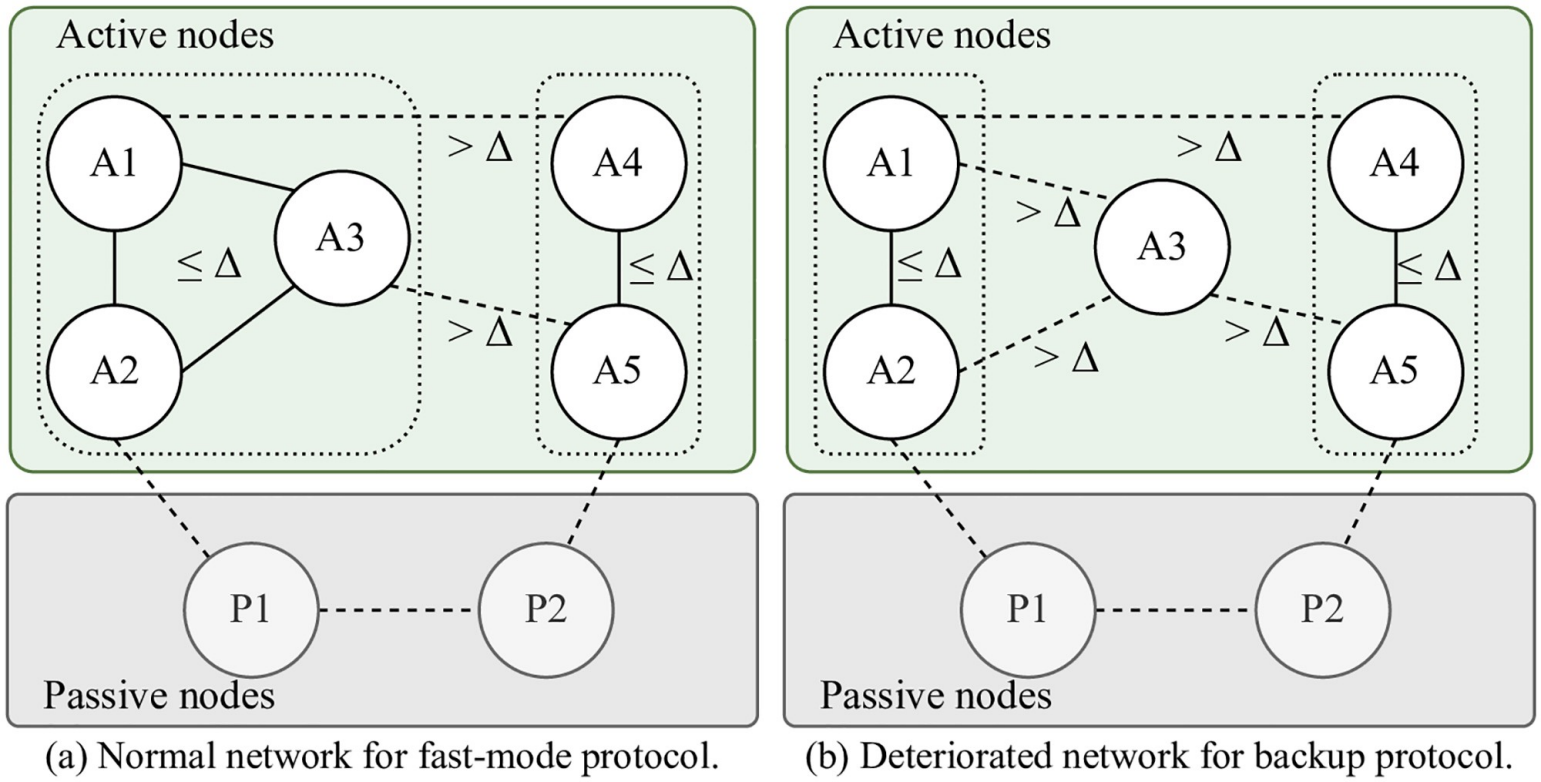
Network conditions for fast-mode and backup protocols.

For the execution of our fast protocol, we assume a *weakly synchronous* network condition, wherein the majority of active nodes can communicate within a known upper bound Δ. As illustrated in Fig 1, the network comprises active and passive nodes. Fig 1(a) depicts the network conditions for the execution of the fast-mode protocol. Active nodes A1, A2, and A3 form a synchronous communication group, and A4 and A5 constitute another synchronous group whose communication delay is smaller than Δ. In this case, the largest synchronous group consists of three active nodes, that is, the majority of active nodes. Therefore, a consensus can be achieved using the fast-mode protocol. Fig 1(b) depicts two synchronous groups: {A1, A2} and {A4, A5}. Their group size is two, which is less than that of the group with majority of the active nodes. Therefore, the fast-mode protocol can no longer maintain its liveness, and the consensus mode returns to the backup protocol.

To confirm the validity of the blocks, we used threshold BLS signatures as a voting mechanism. Notably, each node holds a pair of keys, i.e., a private key used to generate a signature share as a vote regarding the validity of the block and a public key that can verify the signature. For a threshold BLS signature with threshold t, any valid signatures can be combined into an aggregated signature. This aggregated signature possesses the properties of uniqueness and verifiability and is generated based on a non-interactive method. Thus, the aggregated signature can be used both as a proof of block validity and as a random seed ${\text{Rnd}}_{e}$ for each round and epoch. The validity of the aggregated signature can be verified using the group public key generated through the DKG protocol.

As stated, our protocol comprises two subprotocols: a fast protocol and a backup protocol. Each subprotocol has a different consensus committee. Therefore, we generate different group public keys for both subprotocols. We assume that the system comprises n nodes, among which m are active. For the backup protocol, $\left(x_{1},x_{2},...,x_{n}\right)\overset{\left(2f+1,n\right)}{\to }x_{B}$, ${\text{pk}}_{B}=g^{x_{B}}$, where $x_{i}$ denotes the private key of node i, and g denotes the generator of the multiplicative group. Similarly, for the fast protocol, $\left(x_{1},x_{2},...,x_{m}\right)\overset{\left(f+1,m\right)}{\to }x_{F}$, ${\text{pk}}_{F}=g^{x_{F}}$. Correspondingly, ${\text{pk}}_{B}$ and ${\text{pk}}_{F}$ are the group public keys of the backup and fast protocols, respectively, which are used to verify the aggregated signatures generated during the consensus processing of the two subprotocols.

### Protocol overview

**Fig 2:**
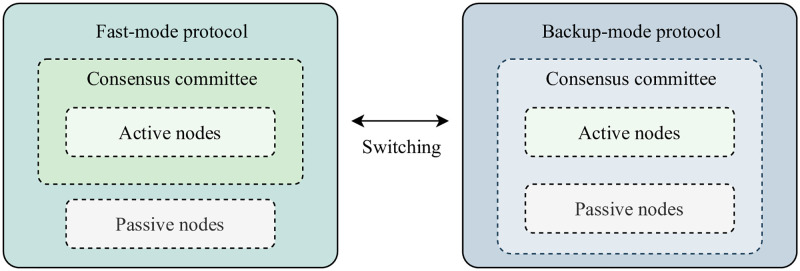
Consensus committee formation for fast-mode and backup-mode protocols.

As depicted in Fig 2, different consensus committees are formed for the fast and backup modes. The nodes in the system play two roles: active and passive. At the beginning of each epoch, the roles are randomly assigned according to the pseudo-random permutation $\pi_{e}$ generated by the epoch random seed ${\text{Rnd}}_{e}$. To further elaborate, in $\pi_{e}$, nodes with sequence numbers 1,2,...,m are active nodes, and nodes with sequence numbers m+1,m+2,...,n are passive nodes. Correspondingly, the active nodes constitute the consensus committee of the fast protocol, and all nodes in the system, including both active and passive nodes, constitute the consensus committee of the backup protocol. For consensus processing, the fast-mode protocol is run by default when the population of well-behaving active nodes is sufficient to constitute a consensus committee for the default mode. When the fast-mode protocol fails to achieve a consensus owing to asynchronous communications among the active nodes, the consensus protocol operation switches to the backup mode, where passive nodes participate in the execution of the protocol, along with the active nodes. Owing to the addition of the backup protocol as a complement to the fast-mode protocol, the agreement protocol can avoid stalling, which results from unfriendly network conditions.

**Fig 3:**
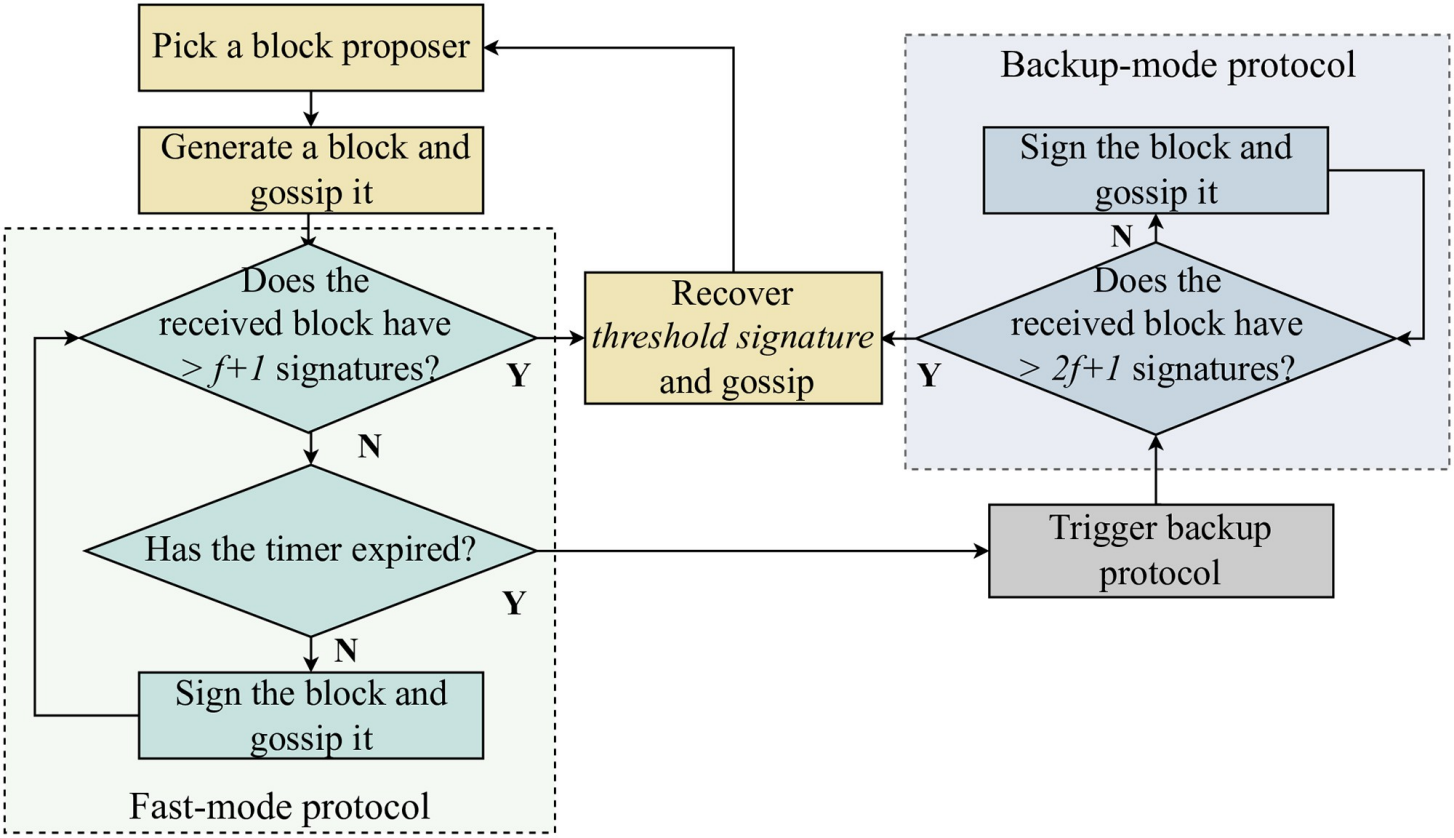
Overall operation of Flexico dual-mode protocol.

The consensus protocol proceeds through a sequence of epochs, where each epoch is subdivided into an increasing number of rounds. We select a block proposer from among the active nodes during each round and let the proposer publish a block. As illustrated in Fig 3, a consensus is achieved using either fast- or backup-mode protocols. The consensus process begins with a fast protocol by default. After generating the block, the proposer signs it and distributes it among other nodes, along with the signature through the gossip protocol. Thereafter, the block proceeds to the verification procedure, which is essentially the process of aggregated signature generation. An honest node is expected to sign the block and gossip it out along with all previously collected signatures. When the block successfully collects the signatures of f+1 active nodes, any node can run the recovery algorithm of the threshold BLS signature to generate the aggregated signature $\sigma_{r,F}$. This signature can be verified by the previously generated group public key ${\text{pk}}_{F}$ and used as a random seed to select the block proposer for the next round. At this point, the fast protocol completes the round of consensus processing. If the block fails to collect the signatures of f+1 active nodes before timeout, the system switches to the backup mode to continue consensus processing. After the switching of the operation mode, the passive nodes join the consensus committee and continue consensus processing for the current round. Unlike the fast protocol, when running the backup protocol, passive nodes also participate in the block verification process, that is, passive nodes can sign the block, providing a vote on the block validity. When the block collects 2f+1 signatures from active nodes and passive nodes, similar to the fast protocol, any node can run the recovery algorithm of the threshold BLS signature to generate the aggregated signature $\sigma_{r,B}$. This signature can be verified by the previously generated group public key ${\text{pk}}_{B}$ and used as a random seed to elect the block proposer for the next round. At this point, the backup protocol completes the current round of consensus processing, and the system automatically switches back to the default fast protocol for consensus processing in the next round.

For protocol safety, we allow every participant to vote once during each round, and the final signature generated based on the collected votes proves the finalization of the proposed block. Owing to synchronous communication among the majority of active nodes, a mode-switching message can be sent after timer expiration to recruit passive nodes that are eligible to participate in the voting process of the backup protocol. Notably, our backup protocol continues the block verification procedure during the current round using all the signatures collected by the default fast-mode protocol, thereby avoiding costly view changes.

### Fast-mode protocol

As the default operation for consensus processing, the fast-mode protocol consists of four primary components.

1. A distributed randomness generation scheme: This scheme enables participants to jointly produce an output that is verifiable and unpredictable across the system. This randomness generating component is built based on the threshold BLS signature generated during the previous round and is determined using Eq 1
  $$\begin{array}{c} {\text{Rnd}}_{r+1}=H({\text{Rnd}}_{r}\left|\right|\sigma_{r}) \end{array}$$ (1)
  where ${\text{Rnd}}_{r+1}$ denotes the randomness for the next round, H denotes a hash function, ${\text{Rnd}}_{r}$ denotes the randomness used in the current round, and $\sigma_{r}$ denotes the threshold signature generated during the current round, which can be either $\sigma_{r,F}$ or $\sigma_{r,B}$. Throughout the process of randomness generation, the threshold signature $\sigma_{r}$ is unpredictable, and the hash function H is verifiable. In addition, the calculation is non-interactive. Thus, participants can independently generate correct and identical randomness, and the results are thereby verifiable and unpredictable.
2. A block proposer selection component based on the generated random seed: A legal block proposer is chosen from a list of active nodes, that is, a list of public keys ordered based on round randomness. The block proposer position Bpp, which indicates the position of its public key in the list, is computed using Eq 2
  $$\begin{array}{c} \text{Bpp}={\text{Rnd}}_{r}\;\text{mod}\;\left(m-1\right) \end{array}$$ (2)
  where m denotes the number of active nodes. Owing to the distributed round randomness generation procedure, the operation of the block proposer selection is unbiased and unpredictable. Moreover, similar to randomness generation, the participants independently determine the position of the block proposer. The Bpp selection operation produces consistent results across the network, ensuring that the validity of the proposed block can be verified using the signature of the block proposer.
3. A block verification process collecting signature shares for the proposed block: Block verification is executed by exchanging signature shares. We utilize a gossip protocol to propagate the blocks and signatures throughout the network, thereby reducing the communication complexity. The block proposer randomly selects log(m) nodes to gossip the proposed block and initiate block verification. When a node receives the proposed block, it checks its validity and attaches its signature as a vote for the block. The signature share $\sigma_{r,i}$ of node i for the current round r is generated using its private key $x_{i}$. The signature share is computed using Eq 3
  $$\begin{array}{c} \sigma_{r,i}=H(r||B_{r}{)}^{x_{i}} \end{array}$$ (3)
  where H denotes a hash function, r denotes the current round number, and $B_{r}$ denotes the proposed block. This received block is then gossiped with all the gathered signatures. The block verification procedure is repeated until the block acquires a sufficient number of signatures to achieve valid approval.
4. A decentralized threshold signature recovery scheme to generate a block finalization proof: When receiving a block with a sufficient number of signatures attached, any participant can run $\text{Recover}(\sigma_{r,1},...,\sigma_{r,f+1})$ to compute the aggregated threshold signature $\sigma_{r,F}$ for the current round. For this, the signature shares $\sigma_{r,1},...,\sigma_{r,f+1}$ must be provided by f+1 different active nodes, after which the Recover function computes the “Lagrange interpolation” for the signatures. Moreover, the threshold signature $\sigma_{r,F}$, which proves that a sufficient number of active nodes have voted for the validity of the block, is sufficient to confirm block finalization. The recovered threshold signature can be verified efficiently using the corresponding public key. Consequently, threshold signature recovery can be conducted in a decentralized and easy-to-verify manner.

**Fig 4:**
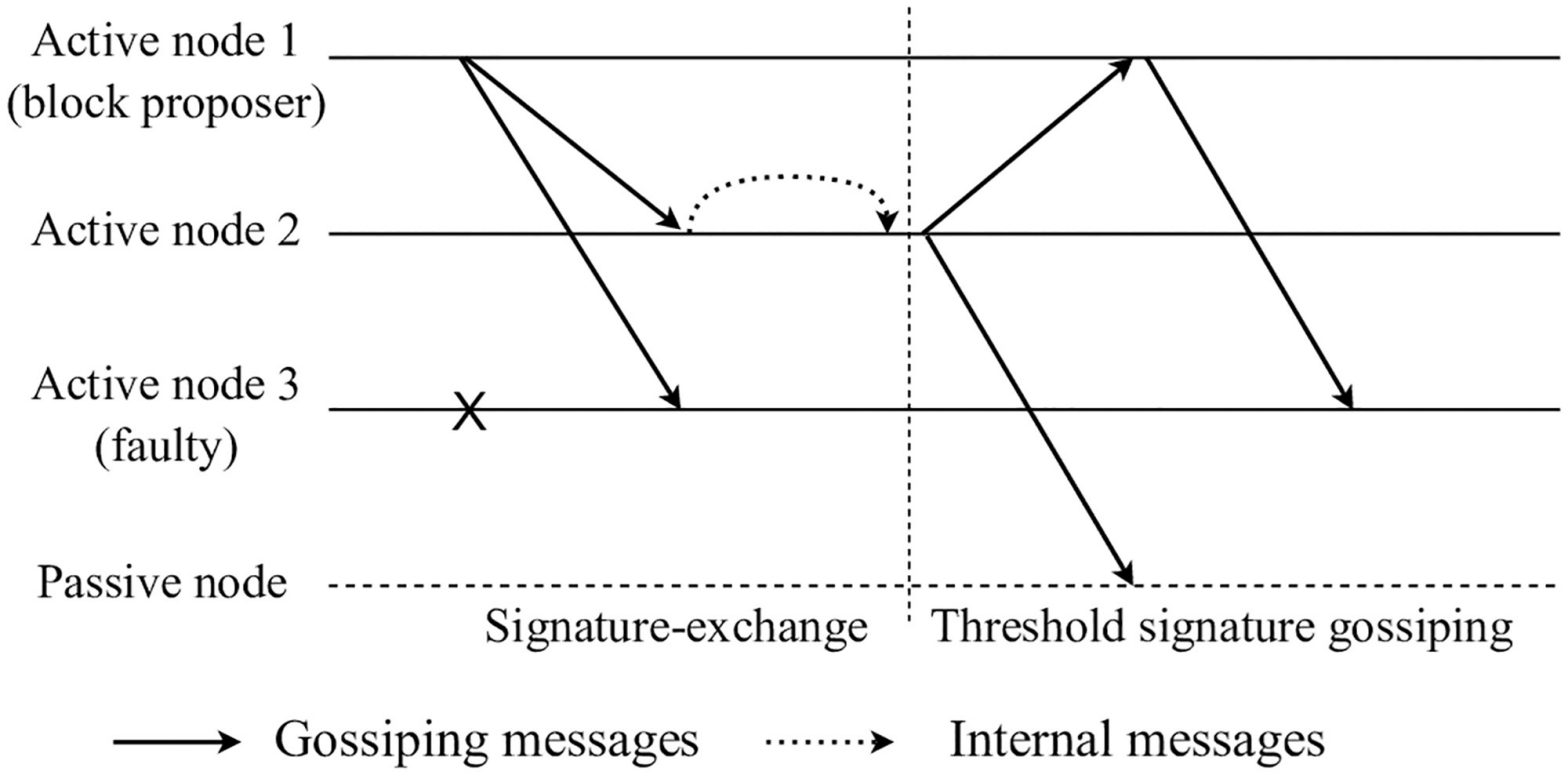
Message flow for *n* = 4, *f* = 1.

The message flow during a fast-mode protocol operation is illustrated in Fig 4. The consensus network contains four nodes, i.e., three active nodes and one passive node. Active node 1 is selected as the block proposer, active node 3 is faulty, and the passive node is not involved in the consensus processing of the fast mode. During the signature-exchange period, the block proposer randomly selects log(m) nodes to send the block proposal and signature. Upon receiving the proposal, active node 2 tests the validity of the block and attaches its signature share. With f+1 signatures appended to the block, the protocol can recover the threshold signature confirming block finalization. Specifically, active node 2 recovers the finalization proof and initiates threshold signature gossiping by randomly selecting log(n) nodes to forward the block with the recovered signature. Thus, the finalized block is propagated across the network. Consequently, the passive node states are also updated according to the finalized blocks. Owing to the use of a threshold signature scheme and gossip communication pattern, our protocol can minimize the communication cost associated with consensus processing.

### Operation mode switching

The network conditions required to run our fast-mode protocol are much easier to achieve than those of existing dual-mode protocols. However, the operation mode should switch to the backup-mode protocol if the default protocol is stuck owing to asynchronous communication between the active modes. In this case, a backup protocol should be triggered to take over the ongoing consensus process. Thus, a careful design of this operation-mode switching mechanism is required. For this, we introduce an operation-mode switching scheme for mode transition in consensus execution. According to the switching operation, active nodes initiate an operation-mode transition by firing a WakeUp message. This message has several components, ${<\text{WakeUp},B_{r-1,\sigma_{r-1}}>}_{\sigma_{i}}$, where $B_{r-1,\sigma_{r-1}}$ denotes the block finalized in the immediately previous round with a valid threshold signature $\sigma_{r-1}$, and $\sigma_{i}$ denotes the signature of message sender i. The pseudocode for operation-mode switching is presented in Alg 1. Upon receiving the threshold signature for the block in the previous round, a node calculates the randomness of the next round and sets a timer. If the timer goes off before receiving the current round block with a valid threshold signature, the node broadcasts a WakeUp message across the network. As the gossip propagates, all active and passive nodes append their signature shares to the WakeUp message. The collected signatures are then recovered into a threshold signature, proving the validity of the WakeUp message.

**Algorithm 1:** Operation mode switching

// <*WakeUp*> message initiation;

**while** *true* **do**

 **if** *isReceived*($B_{r,\sigma_{r,F}}$) *before timeout* **then**

  Reset the timer;

 **else**

  Gossip($<WakeUp{>}_{\sigma_{i}}$);

// <*WakeUp*> message confirmation;

**while** *!confirmed* **do**

 <*WakeUp*> = RcvNewInputs();

 // Check if *B*_*r*_ is finalized;

 **if** *isFinalized*(*B*_*r*_) **then**

  Send back($B_{r,\sigma_{r,F}}$);

  break;

 **if** *count*(*sigShares*) > 2*f* + 1 **then**

  *σ*_*w*,*r*_ = RecoverGroupSig(sigShares);

  Gossip($<WakeUp{>}_{\sigma_{w,r}}$);

  Confirm(<*WakeUp*>) = true;

 **else**

**Fig 5:**
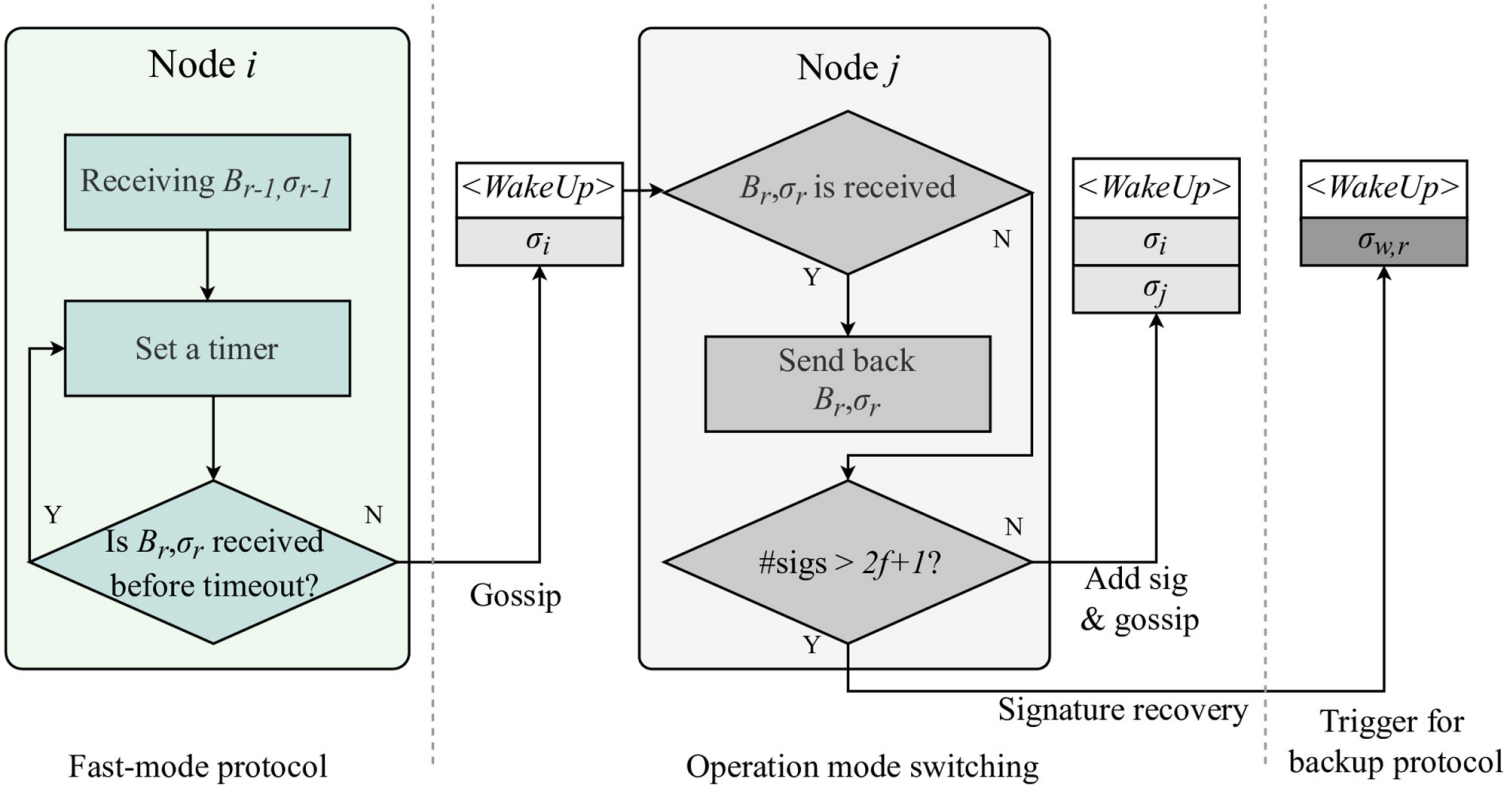
Protocol switching processing.

  sigShares = AppendOwnSignature(<*WakeUp*>);

  Gossip(<*WakeUp*>, sigShares);

Fig 5 illustrates operation-mode switching. Herein, a node initiates mode switching by forwarding a WakeUp message if the finalized block of the current round is not received before timeout. In this scenario, if a node has already received block $B_{r}$ with a valid threshold signature $\sigma_{r,F}$, in response to the WakeUp message, it sends back the finalized block to the sender node. The sender node then returns to the default protocol operation by terminating the mode-switching execution. Otherwise, the node gossips out the WakeUp message with its signature added, which continues the mode-switching execution. Similar to block finalization, WakeUp messages are considered verified when they collect 2f+1 signature shares to indicate that 2f+1 correct nodes are ready to form a consensus committee for backup protocol execution. The signature shares are then recovered into a threshold signature *σ*_*w*,*r*_, which signifies the verification of the WakeUp message. During the mode-switching procedure, the consensus-processing mode can return to the default protocol in the middle, where it can be determined from a valid $B_{r,\sigma_{r,F}}$ receipt, indicating block finalization. After verification of the WakeUp message, the consensus-processing mode changes to a backup protocol operation.

### Backup protocol

After passive nodes locate their positions in the consensus committee, the backup protocol assumes responsibility of consensus processing under partially synchronous conditions. During the backup protocol operation, $<\text{WakeUp}{>}_{\sigma_{w,r}}$ is attached to the block in question, and the block continues to propagate across the network with all collected signature-share votes. Passive nodes participate in the execution of the backup protocol by attaching signature shares as their votes for the block. The threshold signature recovery for the current block is based on all the signature shares collected from the active and passive nodes. Note that passive nodes can only sign a WakeUp message or a proposed block with a valid WakeUp message attached. If a passive node receives a block without a WakeUp message, the node ignores it.

The backup protocol is described in Alg 1. After receiving a WakeUp message with valid threshold signatures, passive nodes participate in the protocol execution together with active nodes. Any node that obtains both the verified WakeUp message and the valid proposed block can propagate two messages together to launch the block verification process of the backup protocol. Similar to the default operation, the gossip protocol is employed for the block verification procedure to propagate the proposed block and signature shares across the network. Both active and passive nodes attach their signatures to the proposed block as votes for block validity. The block verification process is repeated until the block obtains a sufficient number of approvals. Compared with the default protocol, which requires a threshold of f+1 signatures, the backup protocol requires 2f+1 signature shares to recover the threshold signature during the block verification process. After successful recovery of the threshold signature, the proposed block is finalized. The signature is then utilized as a random seed for block proposer selection in the subsequent round. At this point, the backup protocol execution is considered complete. As soon as they receive the threshold signature, passive nodes update their states and reset the timers to quit the consensus committee. More specifically, passive nodes no longer participate in consensus processing until they wake up again in response to a WakeUp message and become a part of the consensus committee. The operation mode then reverts to the default protocol from the subsequent round.

### Checkpoint protocol

To prevent adaptive adversaries from attacking the blockchain network, we re-shuffle committee members during every epoch by generating a new random permutation to reconstruct the consensus committee. Consequently, the node type may change during consensus voting. For example, a passive node in the previous epoch can be listed as an active node in the current epoch. To ensure protocol safety in the forthcoming epoch, the states of all participants must be updated to be consistent with the others, prior to changing their roles. Thus, we employ a checkpoint protocol to synchronize the states of all nodes at the end of each epoch.

**Algorithm 2:** Backup protocol

**while** *!finalized* **do**

 // ** The flag for the validity of *WakeUp* message;

 legal*WakeUp* = fault;

 // ** The flag for the validity of the proposed block;

 legalBlock = fault;

 sigShares, groupSig, *B*_*r*_ = RcvNewInputs();

 // ** Check if the *WakeUp* mesage is verified with 2*f* + 1 signatures;

 **if** *isVerified*(*WakeUp*) **then**

  legal*WakeUp* = true;

 // ** Check if the block is generated by the selected proposer;

 **if** *isValid*(*B*_*r*_) **then**

  legalBlock = true;

 **if** *count(sigShares)* > 2*f* + 1 **then**

  *σ*_*r*,*B*_ = RecoverGroupSig(sigShares);

  Gossip($B_{r,\sigma_{r,B}}$);

  finalized = true;

 **else**

  sigShares = AppendOwnSignature(*B*_*r*_);

  Gossip(*B*_*r*_, sigShares);

The checkpoint protocol is executed by all system nodes, including active and passive nodes, where threshold signature recovery requires 2f+1 signature shares. Unlike regular proposed block-packing transactions, checkpoint blocks carry a state digest instead. Moreover, unlike the regular proposed blocks that rely on a valid WakeUp message to obtain passive node signature shares, passive nodes now verify checkpoint block validity by checking the round number of the block, which is divisible by a pre-known value, e.g., 1024. Owing to the use of a fixed round number, all nodes in the system can attach their signature votes after receiving a valid proposed checkpoint block. Therefore, the checkpoint block can be propagated throughout the network to obtain sufficient signatures for threshold signature recovery.

The checkpoint block finalization proceeds as follows. Similar to regular block verification processing, a proposer is chosen to propagate the checkpoint block proposal represented by the tuple $<e,r,p,d>\sigma_{\text{Bpp}}$, where e denotes the epoch number, r denotes the round number of the block, p denotes the hash pointer to the previous block, $\sigma_{\text{Bpp}}$ denotes the signature of the block proposer, and d denotes the digest of the state. The block proposer spreads the block using the gossip protocol. Upon receiving the checkpoint block proposal, a node attaches its signature as a vote and gossips out the received block if all information is valid. Similar to regular proposed blocks, the threshold signature confirms block finalization. In addition, the recovered signature is used as a source of epoch randomness to obtain a permutation for node identities that engage in the subsequent epoch.

## Fast-mode protocol liveness analysis

Similar to other dual-mode consensus protocols, the fast-path component of our protocol is the key aspect for enhancing its efficiency. The higher the probability of a successful execution to maintain the safety and liveness of the protocol, the better the protocol efficiency. In this section, the probability of successful execution of our fast-mode protocol is analyzed, and the results are compared with those of SBFT, which is a representative dual-mode consensus protocol. We assume that consensus protocols operate in a network with n nodes, where ρ is the probability that the communication delay between two nodes is lower than Δ, that is, the upper bound of the communication delay in a synchronous network.

Notably, protocol liveness relies on network synchrony. For the fast-path protocol of SBFT, maintaining liveness requires a purely synchronous network, which implies that the communication delay between any two nodes must be lower than Δ. Given a network with size n, the total number of connections between nodes is calculated using the combinations ${(}_{2}^{n})$. The probability p of a successful consensus execution through the fast path of SBFT is calculated using Eq 4

$$\begin{array}{c} p=\rho^{{(}_{2}^{n})} \end{array}$$ (4)

By contrast, our fast-mode protocol operation requires a smaller synchronous communication group of active nodes in the network to maintain liveness. Considering the same network size n, the number of active nodes m is computed using Eq 5, and the total connections between active nodes are ${(}_{2}^{m})$. For the fast mode of Flexico, more than m/2 active nodes are required to communicate synchronously. Accordingly, the probability p of a successful agreement is calculated using Eq 6

$$\begin{array}{c} m=\frac{2n+1}{3} \end{array}$$ (5)

$$\begin{array}{c} p=\sum_{i={(}_{2}^{m/2+1})}^{{(}_{2}^{m})}\rho^{i}\times {\left(1-\rho \right)}^{{(}_{2}^{m}-i)} \end{array}$$ (6)

**Fig 6:**
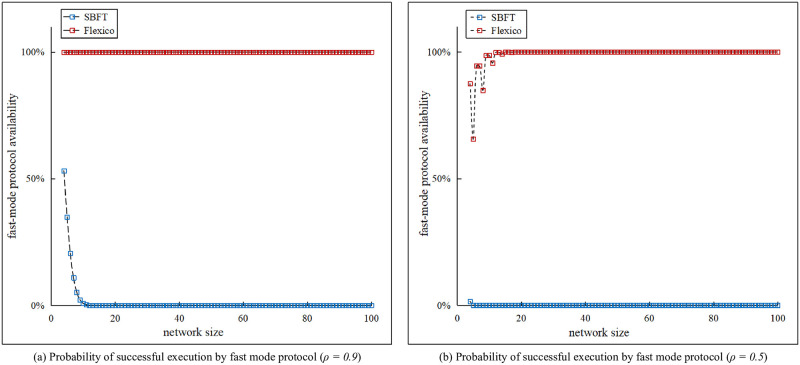
Analysis of fast-mode protocol availability against different network sizes.

Fig 6 compares the results of the fast-mode operations for network sizes ranging from 4 to 100 nodes under different assumptions of ρ. In the case of the SBFT protocol, even for ρ=0.9, the successful execution probability of the fast-path protocol decreases dramatically from 53% to 0 when the network size is greater than 10 nodes. This situation worsens when we consider ρ=0.5, where it becomes nearly impossible to reach an agreement through the fast-path protocol. However, our protocol can always maintain a high success probability of approximately 100% for ρ=0.9. For ρ=0.5, the probability of a successful execution fluctuates in small networks. However, as the network size increases, the fast-mode protocol attains and maintains a high successful execution probability.

## Performance evaluation

In this section, an evaluation of the Flexico protocol based on a simulation is presented, along with an analysis of the results. Note that the primary objective of our evaluation was to analyze the viability and performance of our protocol under different network environments.

### Experimental setup

The tests were conducted using a system equipped with a 2.80 GHz Intel(R) Core(TM) i5-8400 CPU and 8 GB RAM. We implemented our consensus protocol on top of the Cothority framework in Golang. One of the primary goals of this evaluation study was to test whether our protocol experienced severe performance degradation while switching from the default to the backup protocol. The performance of the Flexico protocol was evaluated in terms of the throughput, number of transactions per second (TPS), and latency, that is, the time required to reach a consensus. To demonstrate the effectiveness of our approach, we compared our proposed protocol with other prominent approaches, including PBFT, SBFT, and Concordia. The experiments were conducted using the same testbed under identical restrictions.

### Consensus performance

We evaluated the performance of our consensus protocols in terms of the latency and TPS metrics. Notably, the latency denotes the time required to complete an entire round of a consensus protocol. This metric is primarily affected by the number of nodes participating in consensus processing and the block size. TPS, the evaluation standard for measuring the throughput of the protocol, can be calculated based on the block size and the resulting consensus latency.

**Fig 7:**
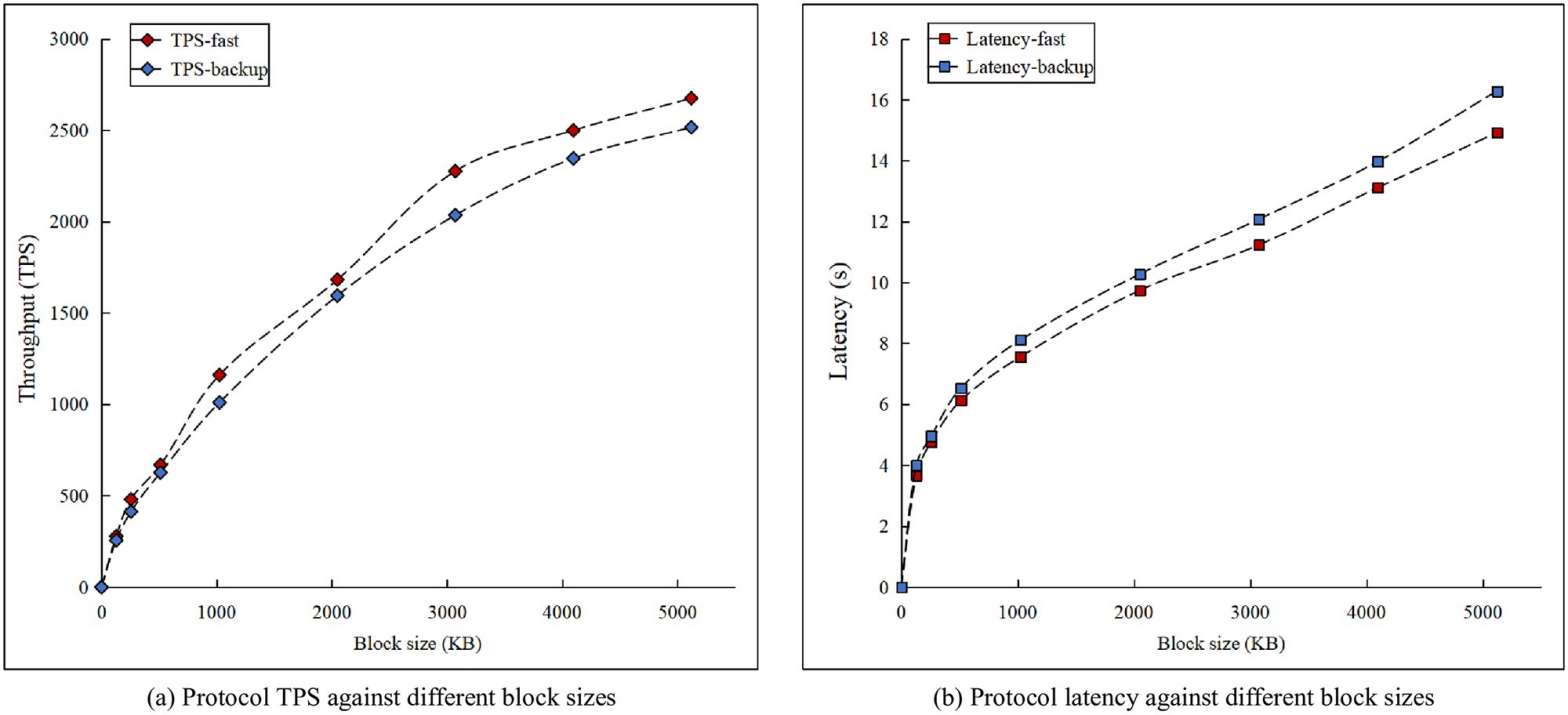
Protocol performance for different block sizes.

For this experiment set, we evaluate the performance of our fast-mode and backup-mode protocols. We create a fixed-size network of 100 consensus nodes and run the protocols with block sizes ranging from 128KB to 5MB. As shown in Fig 7, consensus latency increases with the block size growing. The increasing pattern is similar between fast and backup protocols since both feature the same signature share collection. However, with passive nodes in a dormant state during default operation, the consensus committee size is smaller for fast-mode protocol than for backup protocol. Owing to the efficient block verification procedure, the latency does not significantly increase after the operation mode switches from the fast protocol to the backup protocol. Consequently, compared to the fast protocol, the backup protocol only increases the latency and TPS by 10% on average. The default and backup protocols demonstrate a latency of approximately 10 s for block sizes of up to 2 MB among the 100 participants.

### Consensus latency comparison

**Fig 8:**
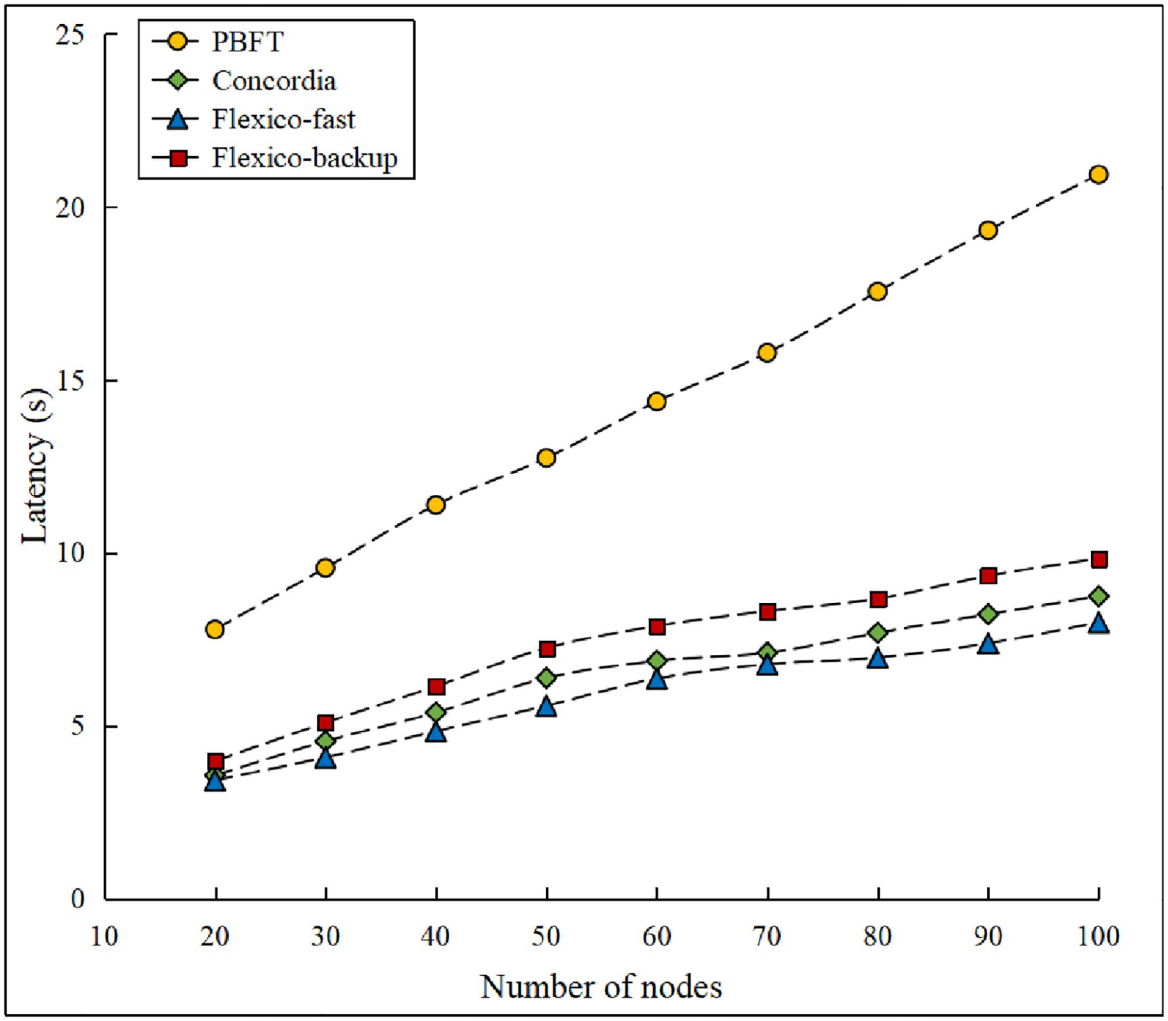
Consensus performance comparison.

Consensus latency is one of the most critical metrics for evaluating a consensus protocol. During the experiment, we fixed the block size to 1 MB and ran protocols with varying network sizes ranging from 20 to 100 nodes. The latency results for Flexico, PBFT, and Concordia are plotted in Fig 8. Because PBFT represents a classical consensus protocol for Byzantine environments, we chose it as our baseline protocol. In addition, our protocol was derived from Concordia.

As shown in Fig 8, the consensus latency increased with the number of nodes in the system. As expected, the PBFT protocol exhibited the highest latency owing to its all-to-all broadcast pattern. The fast-mode protocol of Flexico achieved the best consensus latency owing to the smaller committee size and use of a gossip-based communication protocol, which decreased the communication complexity to O(logm). These results indicate that the fast protocol of Flexico can achieve 70% lower latency than PBFT. The backup protocol of Flexico demonstrated a slight degradation in performance. However, the latency was maintained at an acceptable level. Compared to our previous study on Concordia, which is an efficient synchronous consensus protocol, we did not focus on improving the performance here. However, Flexico significantly improved the availability of Concordia by building a consensus protocol that could process a consensus in both weakly and partially synchronous environments. A comparison of the different consensus protocols is presented in Table 3.

**Table 3:** Flexico and its predecessor consensus protocols.

Protocol	Adversary Tolerate	Communication Model	Communication Complexity	Throughput	Latency
PBFT	*f* < *n*/3	Patially Synchronous	*O*(*n*^2^)	Low	High
Concordia	*f* < *n*/2	Weakly Synchronous	*O*(*n*^2^)	High	Low
Flexico-fast	*f* < *n*/3	Weakly Synchronous	*O*(*log* *m*)	High	Low
Flexico-backup	*f* < *n*/3	Patially Synchronous	*O*(*log* *n*)	High	Low

### Performance comparison with dual-mode protocol

**Fig 9:**
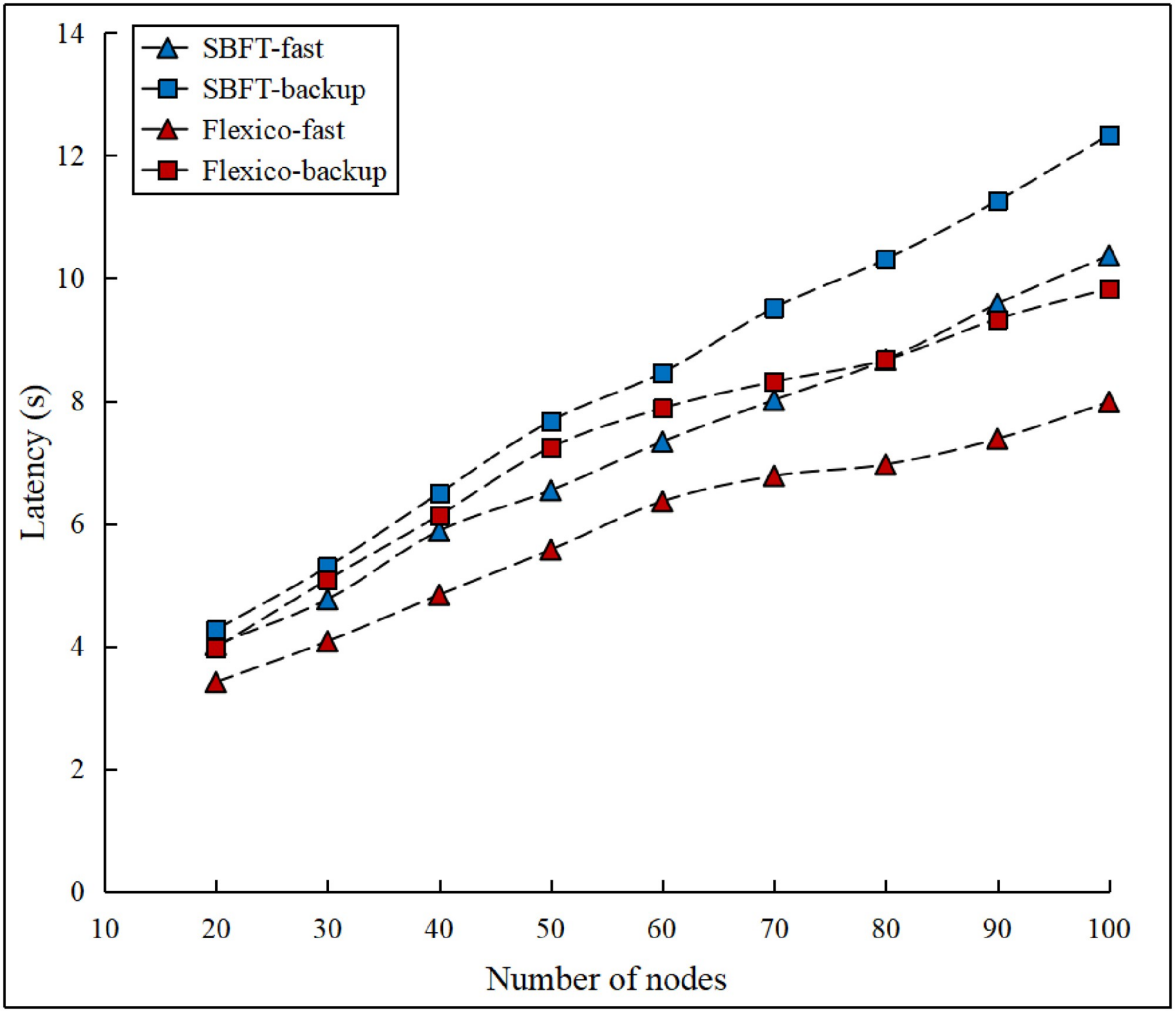
Dual-mode protocol latency comparison.

We ran an additional set of experiments to compare our protocol with the state-of-the-art dual-mode SBFT protocol, which uses the BLS-threshold signature scheme. SBFT uses linear communication between replicas with commit collectors, whereas our approach exploits gossip-based message propagation. The latency results of the fast- and default-mode protocols for SBFT and Flexico are compared in Fig 9.

As shown in the Fig 9, after switching to the backup protocol, the performance degraded for both SBFT and our proposed protocols. However, our proposed fast mode and backup-mode protocols resulted in a lower latency than SBFT. Moreover, owing to different communication patterns, the increased latency originating from the increasing network size exhibited different rates. As expected, in the case of SBFT, the latency increased at a linear rate, whereas the latency of our protocol exhibited a logarithmic growth. Compared to SBFT, the latency of Flexico increased more gradually. As the network size increased, the latency of our backup protocol became smaller than that of the fast protocol of SBFT.

**Fig 10:**
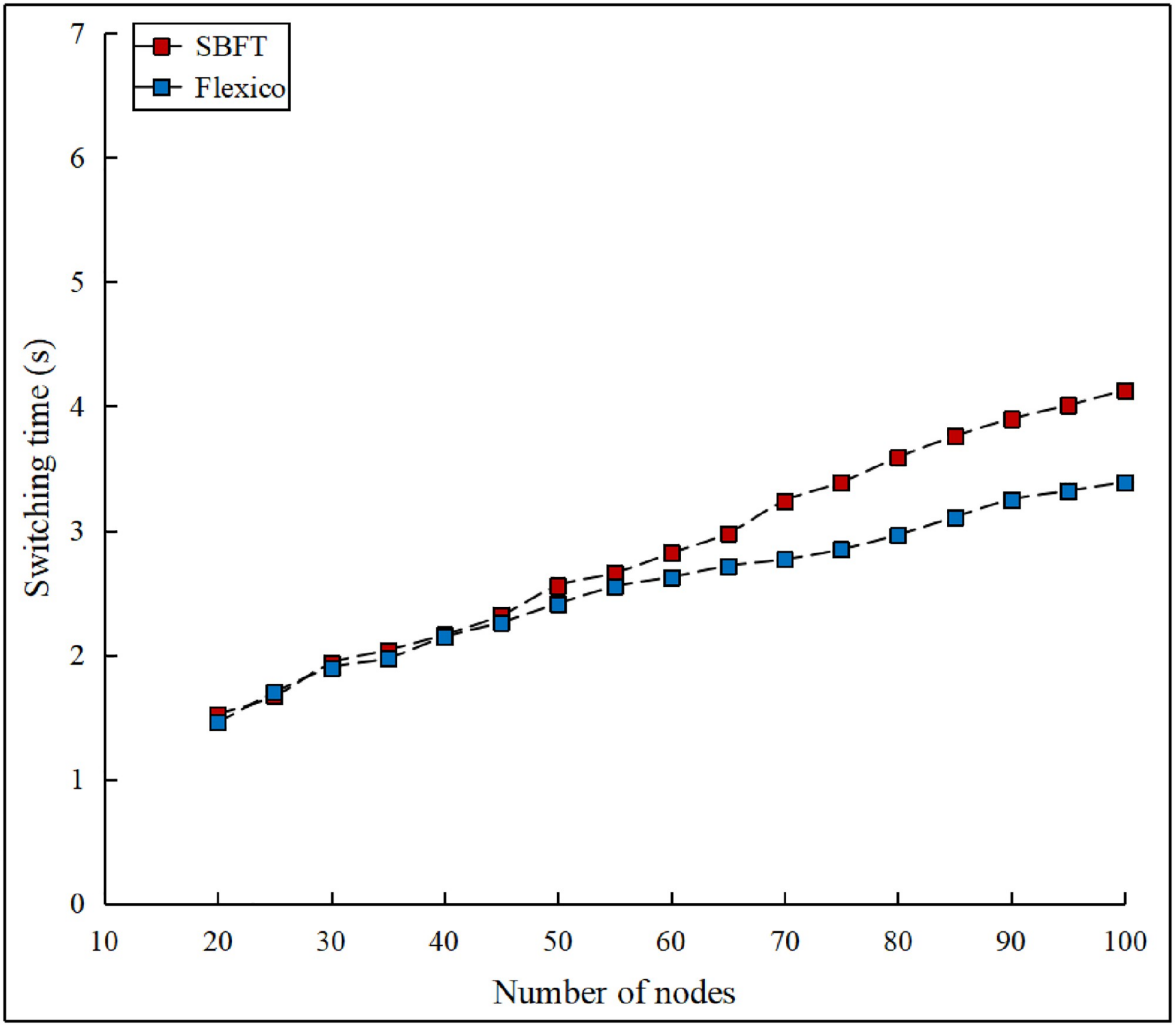
Protocol switching time comparison.

We also compared the switching times between the two modes for the SBFT and Flexico based approaches. To measure the operation-mode switching time, we set the timeout value to a preconfigured value τ value. The obtained results are plotted in Fig 10. The figure indicates that the time required to activate the backup protocol is almost identical for both SBFT and Flexico in a small-scale network. However, the gap becomes more apparent as the network size increases, which can be attributed to message gossiping.

The comparison between Flexico and the state of art dual-mode protocol SBFT is shown in Table 4. Compared with SBFT, our fast-mode protocol can tolerate up to f<n/3 Byzantine adversaries. In addition, our fast protocol can operate in a weakly synchronous network, which allows it to achieve a fairly high usage rate. The fast protocol of the SBFT protocol, by contrast, has an extremely low usage rate owing to the requirement of a pure synchronous network state. In addition, we further reduce the linear communication complexity of SBFT to that of logarithmic communication. Moreover, our protocol does not require a view change when conducting consensus-processing mode switching. These features enable our protocol to achieve better performance.

**Table 4:** Comparison with SBFT protocol.

		**SBFT**	**Flexico**
**Fast-mode**	**Adversary tolerate**	*f* = 0	*f* < *n*/3
	**Communication model**	Synchronous	Weakly synchronous
	**Communication complexity**	*O*(*n*)	*O*(*log* *m*)
	**Utilization**	Very low	Very high
**Backup-mode**	**Adversary tolerate**	*f* < 3/*n*	*f* < 3/*n*
	**Communication model**	Partially synchronous	Partially synchronous
	**Communication complexity**	*O*(*n*)	*O*(*log* *n*)
**View change**		✓	☓

## Related work

Notably, distributed consistency protocols are the focal point of distributed computing systems. Crash fault tolerant protocols requiring 2f+1 nodes in a partially synchronous network are being utilized by several notable systems including Google Chubby and Apache Zookeeper [41, 42]. To deal with Byzantine faults, 2f+1 nodes are required in a synchronous network, whereas 3f+1 nodes are required in a partially synchronous network. Thus, certain BFT protocols such as Synchronous BFT and FlexibleBFT [24, 43] assume synchronous networks for a smaller consensus quorum, which indicates a lower cost for message exchange. However, in reality, pure synchrony cannot be easily obtained, which is one of the reasons why partially synchronous BFT protocols are becoming mainstream. PBFT, the first practical BFT protocol, is widely used in several distributed systems including permissioned blockchain systems. However, the performance of the PBFT protocol is considered problematic owing to the communication complexity of $O(n^{2})$.

Thus, significant effort has been dedicated to improve the efficiency of partially synchronous BFT protocols. To that end, Hotstuff [44] and SBFT protocols [12] turn all-to-all communication model into a linear approach and thus decrease the communication complexity to O(n). Although the protocol execution adds extra steps, the communication cost is lower than that of PBFT. LibraBFT [45] further refines the Hotstuff protocol used in the Libra blockchain [46]. ByzCoin [47] builds a PBFT-style consensus protocol on top of Schnorr’s collective signing protocol, which lowers its communication complexity to O(logn). Similarly, other protocols have proposed consensus schemes based on the BLS-threshold signature technology. The recovered threshold signature is 32 bytes long, which is much smaller than that of the other methods. Although the proposed protocol employs a BLS-threshold signature, unlike protocols that require two rounds of message exchanges, our protocol requires only a single round of communication to achieve block finalization.

A group of protocols including [12–15] add an optimistic sub-protocol for faster consensus processing. add an optimistic subprotocol for faster consensus processing. Specifically, SBFT introduces a fast-path protocol for friendly network conditions comprising nonfaulty synchronous nodes. The protocol returns to the linear PBFT protocol when a fault occurs in the system. Similarly, CheapTiny is an optimistic protocol that resorts to the MinBFT protocol if the network conditions deteriorate [48]. In addition, the CheapBFT protocol is augmented by leveraging trust hardware to prevent equivocation, which reduces the minimum network size from 3f+1 to 2f+1. These dual-mode protocols allow operation-mode switching between optimistic and backup protocols and demonstrate better performance under different system conditions. However, to benefit from a dual-mode operation, the protocols require a nearly ideal condition wherein all nodes in the network are nonfaulty and synchronous. By contrast, the conditions for our default protocol appear relaxed for improved-performance options.

## Future work

With the development in IoT and edge computing technologies, more attention is being paid to the security of IoT data in edge networks. Over the past years, blockchain as a decentralized tamper-proof data store has been hailed as a solid foundation on which to devise technical solutions to the issue. In fact, this study is a part of our long-term project on developing a secure edge platform on top of blockchain layer. In contrast to traditional cloud nodes, it is common that edge nodes and IoT devices are constrained in terms of computation power and communication capability. In this regard, resource-hungry mining algorithms, such as the Proof of Work, are not a good fit to edge network environments. Many blockchain consensus protocols targeting edge computing environments look to committee-based BFT variants. One downside of these protocols is that they require stable networking conditions for good performance. Flexico is designed to perform reasonably well in degenerating network conditions as well, being able to manage a low latency even under unstable network conditions. This opens up a possibility of Flexico as a versatile agreement protocol for IoT edge networks where network instability and communication disruption are more likely. In the future, we plan to develop an edge computing architecture, built on Flexico protocol, capable of securely processing IoT data and automating provisioning and management decisions for the network. Through a case study involving IoT data and edge computing scenarios, we hope to gain deeper insights on the gains and further possibility of Flexico, and accordingly upgrade the protocol.

## Conclusion

This article proposes Flexico, a highly efficient dual-mode consensus protocol whose consensus performance is substantially boosted using a fast-path component. Its backup protocol companion complements fast-mode protocol executions to continue consensus processing even under deteriorated network conditions. Our evaluation results indicate that the default protocol of Flexico exhibits a better performance in terms of the confirmation latency, which is approximately 7s in a network with 100 nodes and a block size of 1 MB. Furthermore, its backup protocol can enhance the liveness of the protocol while achieving a comparable latency of approximately 10s. By exploiting both passive and active nodes for consensus voting, our proposed protocol maximizes its performance gain at a lower communication cost. In addition, switching operations between the two modes can be conducted without expensive view changes, which further adds to the efficiency gain of the protocol. Our protocol can still be improved in several aspects. One particular problem that remains to be addressed is its ability to support large-scale networks. Currently, Flexico can accommodate hundreds of nodes, which seems satisfactory for our target network. If one considers a case of vast IoT networks comprising a huge number of nodes, further research would be needed to enhance the scalability of the consensus protocol.
